# Prognostic impact of myosteatosis in patients with colorectal cancer undergoing curative surgery: an updated systematic review and meta-analysis

**DOI:** 10.3389/fonc.2024.1388001

**Published:** 2024-06-19

**Authors:** Yu-Yao Chang, Bill Cheng

**Affiliations:** ^1^ Division of Colon and Rectal Surgery, Department of Surgery, Changhua Christian Hospital, Changhua, Taiwan; ^2^ Department of Post-Baccalaureate Medicine, College of Medicine, National Chung Hsing University, Taichung, Taiwan; ^3^ Graduate Institute of Biomedical Engineering, National Chung-Hsing University, Taichung, Taiwan

**Keywords:** colorectal cancer (CRC), myosteatosis, prognosis, survival, systematic review and meta-analysis

## Abstract

**Background:**

Colorectal cancer (CRC) is a global health concern, and identifying prognostic factors can improve outcomes. Myosteatosis is fat infiltration into muscles and is a potential predictor of the survival of patients with CRC.

**Methods:**

This systematic review and meta-analysis aimed to assess the prognostic role of myosteatosis in CRC. PubMed, Embase, and Cochrane CENTRAL were searched up to 1 August 2023, for relevant studies, using combinations of the keywords CRC, myosteatosis, skeletal muscle fat infiltration, and low skeletal muscle radiodensity. Case–control, prospective, and retrospective cohort studies examining the association between myosteatosis and CRC outcomes after curative intent surgery were eligible for inclusion. Primary outcomes were overall survival (OS), disease-free survival (DFS), and cancer-specific survival (CSS).

**Results:**

A total of 10 studies with a total of 9,203 patients were included. The pooled hazard ratio (HR) for OS (myosteatosis vs. no myosteatosis) was 1.52 [95% confidence interval (CI), 1.38–1.67); for CSS, 1.67 (95% CI, 1.40–1.99); and for DFS, 1.89 (95% CI, 1.35–2.65).

**Conclusion:**

In patients with CRC undergoing curative intent surgery, myosteatosis is associated with worse OS, CSS, and DFS. These findings underscore the importance of evaluating myosteatosis in patients with CRC to improve outcomes.

## Introduction

Colorectal cancer (CRC) is a worldwide health challenge and is associated with high mortality rates (1, 2). In 2020, CRC accounted for approximately 9.4% of all cancer-related fatalities (3). Although multiple prognostic factors associated with the outcomes of CRC have been identified, recent study has suggested that body composition, particularly myosteatosis, may be associated with CRC outcomes (4).

Myosteatosis refers to the accumulation of fat within muscle tissue, independent of obesity (5). It has been identified as an independent predictor of poor outcomes in different diseases, including cancers (6). Studies have suggested that myosteatosis may be a prognostic factor in patients with CRC (4, 6). Myosteatosis is often associated with the sarcopenia (i.e., reduction in skeletal muscle mass) and has been associated with adverse outcomes in patients with various cancers, including increased treatment-related toxicity, impaired functional status, increased postoperative infectious complications, worse oncological outcomes, reduced quality of life, and diminished overall survival (OS) (4, 7–10). In patients undergoing colorectal surgeries, including those with Crohn’s disease, myosteatosis has been linked to an increased risk of postoperative complications, such as surgical site infections and anastomotic leaks (11). Preoperative myosteatosis has been associated with adverse effect on short- and long-term outcomes in patients with CRC undergoing surgical resection (12).

The most recent systematic review examining the prognostic relevance of myosteatosis in CRC was published in 2020 (13). However, that review was constrained by a limited sample size, which impeded its ability to establish a definitive conclusion. Additionally, its dependence on pre-2020 studies fails to account for recent advancements in care. Since the publication of that review, a number of additional reports have been published, further exploring the relationship between myosteatosis and CRC outcomes. Consequently, there exists a need for an updated systematic review and meta-analysis to offer a more thorough evaluation of the impact of myosteatosis on the prognosis of patients with CRC.

Hence, the primary objective of this systematic review and meta-analysis was to use the most current research results to perform a meta-analysis to determine the impact of myosteatosis on the prognosis of patients with CRC undergoing curative surgery.

## Methods

### Search strategy

This present systematic review and meta-analysis was conducted in accordance with the Preferred Reporting Items for Systematic Reviews and Meta-Analyses (PRISMA) guidelines (14). PubMed, EMBASE, and Cochrane CENTRAL databases were searched for studies published up to 1 August 2023. The keywords used were “colorectal cancer”, “myosteatosis, “skeletal muscle fat infiltration”, “lower skeletal muscle radiodensity”, combined with Boolean operators, and using Medical Subject-Headings (MeSH) terms where appropriate. An example search string used for PubMed was:

(Colorectal cancer) AND (myosteatosis OR “skeletal muscle fat infiltration” OR “low skeletal muscle radiodensity”)

In addition, the reference lists of included studies were hand-searched to identify other potentially relevant studies.

### Selection criteria

This review was performed in accordance with the Population Exposure Comparison and Outcome (PECO) framework. Eligible studies were those investigating patients with stage I–IV CRC undergoing curative intent surgery and in which patients were categorized into two groups based on the presence or absence of myosteatosis by routine abdominal-pelvic computed tomography (CT) within 60 days before surgery. For inclusion, a study had to have reported at least one of the outcomes of interest, including OS, disease-free survival (DFS), and cancer-specific survival (CSS). Case–control, retrospective, or prospective cohort studies were considered for inclusion.

Studies that included patients with double primary cancers, not focused on CRC, or in which patients underwent emergency operations were excluded. In addition, review articles, letters, commentaries, editorials, proceeding research, meeting abstracts, case reports, and personal communications were excluded. Studies in a language other than English were also excluded.

The eligibility of studies identified via the above search and selection strategy was confirmed by two independent reviewers, and a third reviewer was consulted where there was uncertainty regarding eligibility.

### Diagnosis of myosteatosis

Eligible studies were those in which skeletal muscle areas were assessed during preoperative venous-phase CT examination. Muscle density was measured as mean Hounsfield unit (HU) at the cross-sectional muscle area at the L3 level. Myosteatosis was defined as HU < 41 for patients with a body mass index (BMI) < 25 kg/m^2^, and HU < 33 for patients with BMI ≥ 25 kg/m^2^.

### Main outcome measures and data extraction

The primary outcomes of interest were OS, DFS, and CSS. OS was defined as the time between the day of surgery and the day of death due to any cause, or the last follow-up date. DFS was calculated as the period from surgery to the time of relapse or death from non-cancer cause. CSS was defined as the time from surgery to death caused by CRC.

Data extracted from eligible studies included the name of the first author, year of publication, study design, study country, number of patients with and without myosteatosis, mean patient age, sex distribution (% male), mean follow-up time, and outcomes of interest.

### Ethics statement

This systematic review and meta-analysis of published studies neither required nor used raw patient data and private information; therefore, approval of the protocol by the Institutional Review Board (IRB) of the Changhua Christian Hospital and informed consent from study subjects were waived. This review was not pre-registered in a public database.

### Quality assessment

The quality of included studies was assessed using the Newcastle–Ottawa scale (NOS) for cohort studies, as recommended by the Cochrane Non-randomized Studies Methods Working Group (15). The NOS awards a maximum of 9 points to each study, representing 4 points for the adequate selection of cohort participants, 2 points for comparability of the cohort participants based on the design and analysis, and 3 points for adequate ascertainment of outcomes. Quality assessment was performed by two independent reviewers, and a third reviewer was consulted if any uncertainties occurred.

### Statistical analysis

Cox regression models were used to analyze OS, CSS, and DFS as time-to-event measures, and adjusted hazard ratios (HRs) reported by each included study were pooled to obtain summary effects. Heterogeneity among the studies was assessed using the Cochran Q test and the I^2^ statistic. An I^2^ index > 50% indicates the presence of significant heterogeneity, and a random-effects model was used; otherwise, a fixed-effects model was employed. All analyses were two-sided, with a significance level of α = 0.05. To assess the robustness of the results, a sensitivity analysis was performed using the leave-one-out approach. Potential publication bias was assessed via funnel plot asymmetry by Egger’s test. A symmetric, funnel-shaped distribution of data points suggests the absence of publication bias. All analyses were conducted using R Studio software, with the packages “meta”, “dmetar”, and “metafor”.

## Results

### Study selection

**Figure 1:**
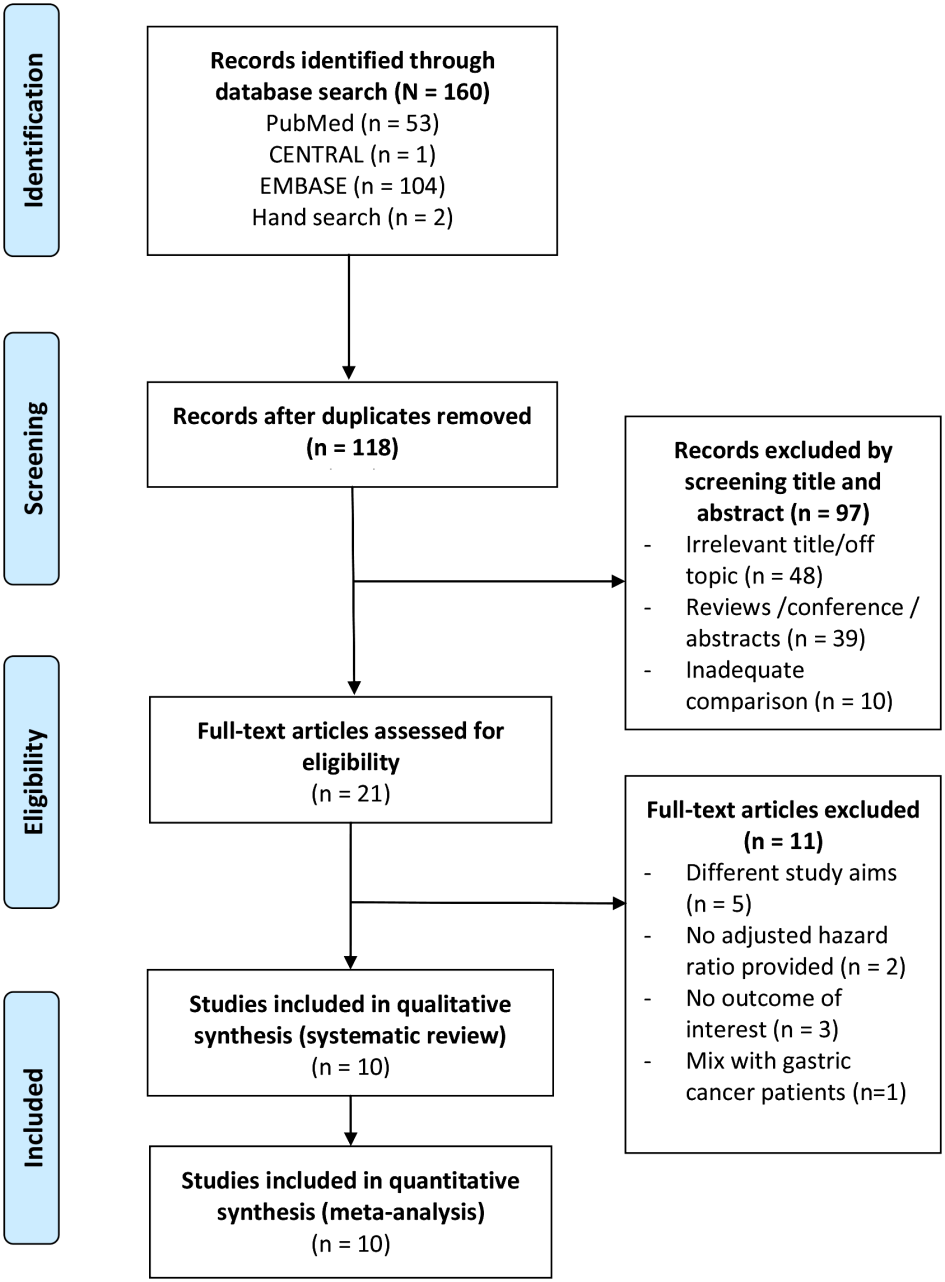
PRISMA flow diagram of study selection.

The PRISMA diagram of study selection process is shown in **Figure 1**. A total of 21 full-text articles were assessed for eligibility, and 11 were excluded. Therefore, 10 studies (16–25) consisting of a total of 9,203 patients with CRC were included in the systematic review and meta-analysis.

### Characteristics of included studies

The characteristics of included studies are summarized in **Table 1**. Only one study was of prospective design (19), and the other were of retrospective design. Four studies were conducted in Asia (16, 17, 22, 24, 25), four in Europe (18, 19, 21, 23), and two in North America (16, 20). The proportion of male patients ranged from 53% to 64%, and the proportion of primary tumors at the rectum ranged from 7% to 60%.

**Table 1:** Characteristics of included studies.

First author and year of publication	Study design	Country	Number of patients		Age (years)	Male (%)	BMI (kg/m^2^)	Primary tumor at the rectum (%)	TNM stage	Length of follow-up (months)	NOS score
			Total	Myosteatosis Yes/No							
Chen et al. (24)	Retrospective	China	921	633/288	Median (IQR): 73 (10)	61	Median (IQR): 22.65 (4.32)	43	I/II/III/IV: 211 (22.9%)/ 374 (40.6%)/ 327 (35.5%)/ 9 (1.0%)	Median: 34	7
Koh et al. (25)	Retrospective	Korea	1,015	842/173	< 70: 702 (69.1%) ≥ 70: 313 (30.9%)	59	Mean: 23.6 ± 3.0 Mean: 22.3 ± 3.2	7	I-II/III/IV: 531/380/104	91.6 (71.1–118.3)	7
Kroenke et al. (16)	Retrospective	USA	3,262	966/2,292	< 50: 432 (13.2%); 50 to < 60: 806 (24.7%); 60 to < 70: 941 (28.8%); ≥ 70: 1,083 (33.2%);	59	18.5 to < 25.0: 156 25.0 to < 30: 912 ≥ 30.0: 385	29	I/II/III: 979/1,030/1,253	6.9 (0–10.9)	8
Park et al. (22)	Retrospective	Korea	727	132/595	≥ 60: 457 (62.9%); < 60: 270(37.1%)	59	< 25: 512 (70.4%); ≥ 25: 215 (29.6%)	29	I-II/III/IV: 384/259/84	98 (70–102)	6
Aro et al. (23)	Retrospective	Finland	222	67/155	≤ 70: 95 (42.8%); > 70: 127 (57.2%)	53	< 18.5: 3 (1.4%) 18.5 to 24.9: 73 (32.9%) 25 to 29.9: 92 (41.4%) ≥ 30: 54 (24.3%)	34	I/II/III/IV: 42 (18.9%)/ 75 (33.8%)/ 73 (32.9%)/ 32 (14.4%)	82 ± 45.3	8
Hopkins et al. (20)	Retrospective	Canada	968	590/376	Mean: 65.8 ± 11.8	61	27.7 ± 5.7	NR	AJCC I/II/III: 100 (10.3%)/ 374 (38.6%)/ 494 (51.0%)	5.2 (0.01–10.25) years	8
Aro et al. (21)	Retrospective	Finland	346	108/238	Myosteatosis: Mean: 74 ± 10.7 No myosteatosis: Mean: 66 ± 10.9	53	Myosteatosis: 26.5 ± 5.3 No myosteatosis: 27.6 ± 4.6	60	I/II/III: 25 (23.4%)/ 45 (42.1%)/ 37 (34.6%)	72 ± 29.7	8
Sueda et al. (17)	Retrospective	Japan	211	101/110	< 65: 104 (49.3%); ≥ 65: 107 (50.7%)	64	< 20: 45 (21.3%) 20 to 24: 121 (57.3%) 25–29: 42 (19.9%); ≥ 30: 3 (1.4%)	35	NR	Median: 57.6	7
van Baar et al. (18)	Retrospective	Netherlands	715	519/196	Mean: 67.7 ± 10.3	62	< 20: 25 (4%) 20 to 24.9: 255 (36%) 25 to 29.9: 316 (44%); ≥ 30: 119 (17%)	32	I/II/III: 210 (29.4%)/ 204 (28.5%)/ 301 (42.1%)	48 (0–119)	6
van Vugt et al. (19)	Prospective	Netherlands	816	293/523	Median: 70	54	NR	NR	NR	Median: 76.5	7

IQR, interquartile range; NOS, Newcastle-Ottawa Scale; NR, not reported; BMI, body mass index; AJCC, American Joint Commission on Cancer.

### Meta-analysis

#### Association between myosteatosis and OS

**Figure 2:**
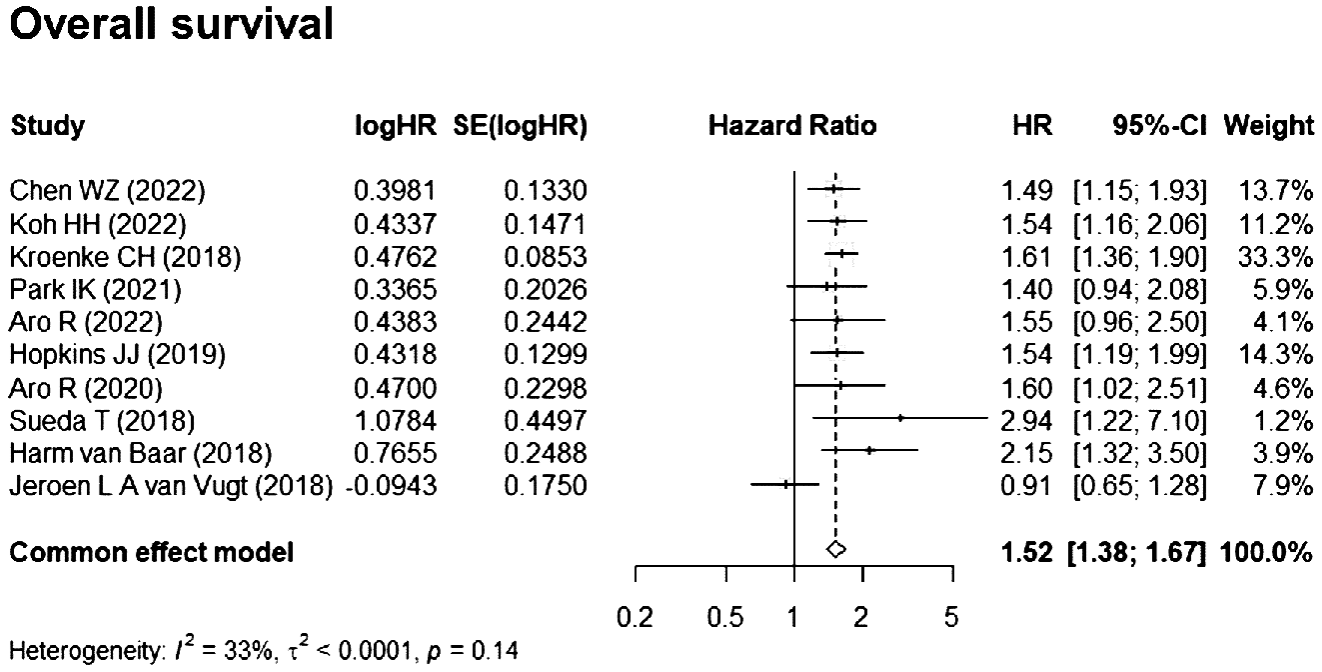
Association between myosteatosis (vs. no myosteatosis) and overall survival (OS).

A total of 10 studies (16–25) reported HRs for the impact of myosteatosis on OS and were included in the meta-analysis. Based on the heterogeneity index (I^2 = ^33%) and Q test (p = 0.14), a fixed-effects model was applied. The pooled HR for OS (myosteatosis vs. without myosteatosis) was 1.52 [95% confidence interval (CI), 1.38–1.67], indicating a statistically significant association between myosteatosis and reduced OS (**Figure 2**).

#### Association between myosteatosis and CSS, DFS

**Figure 3:**
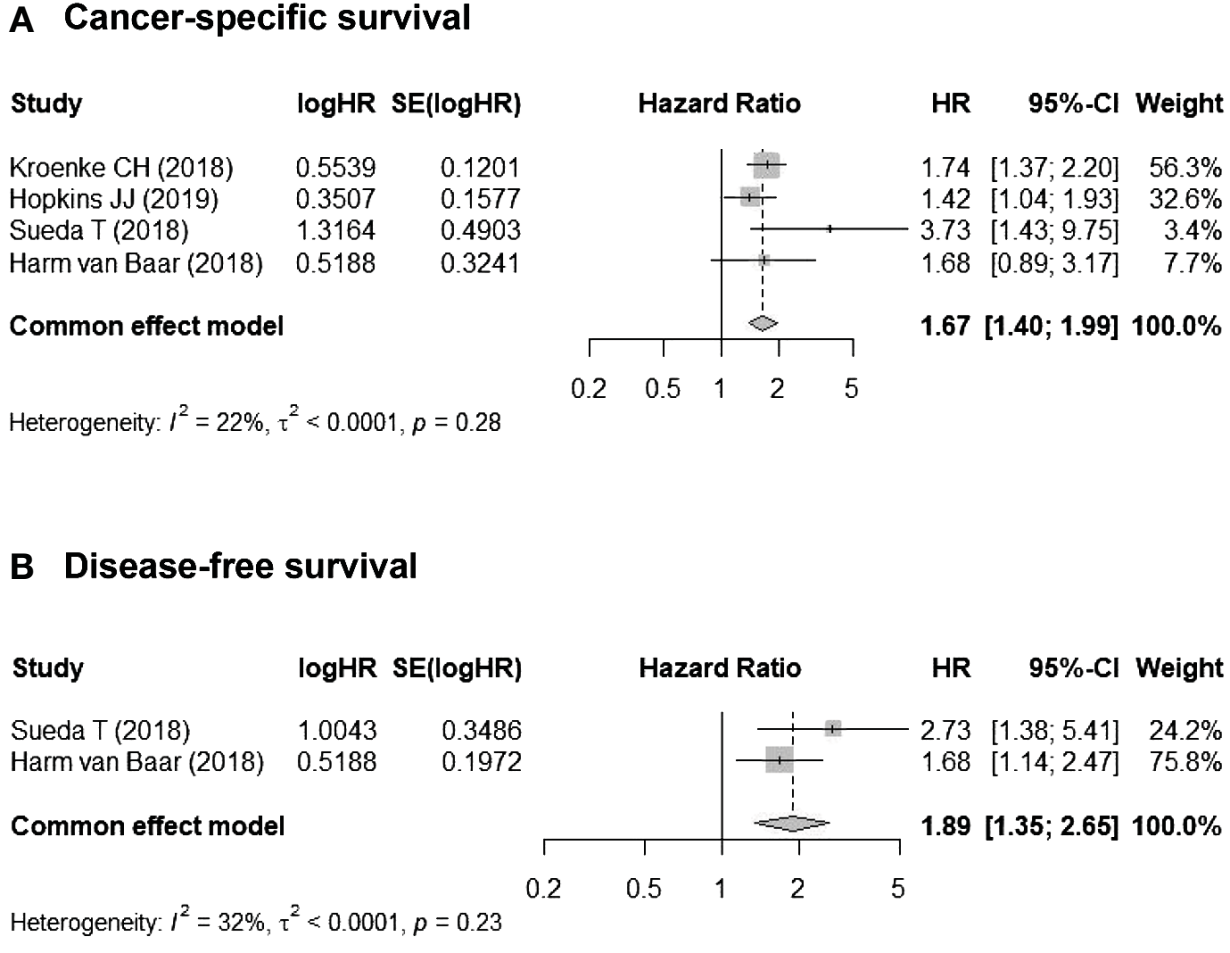
Associations between myosteatosis (vs. no myosteatosis) and **(A)** cancer-specific survival (CSS) and **(B)** disease-free survival (DFS).

Four studies reported CSS (16–18, 20), and two reported DFS (17, 18). The I^2^ was < 40% for all studies, and the Cochran Q was insignificant; thus, fixed-effects models were employed. For CSS, the pooled HR for myosteatosis (vs. no myosteatosis) was 1.67 (95% CI, 1.40–1.99). For DFS, the pooled HR for myosteatosis (vs. no myosteatosis) was 1.89 (95% CI, 1.35–2.65). Taken together, these data indicate that myosteatosis is associated with worse CSS and DFS (**Figure 3**).

### Publication bias assessment

**Figure 4:**
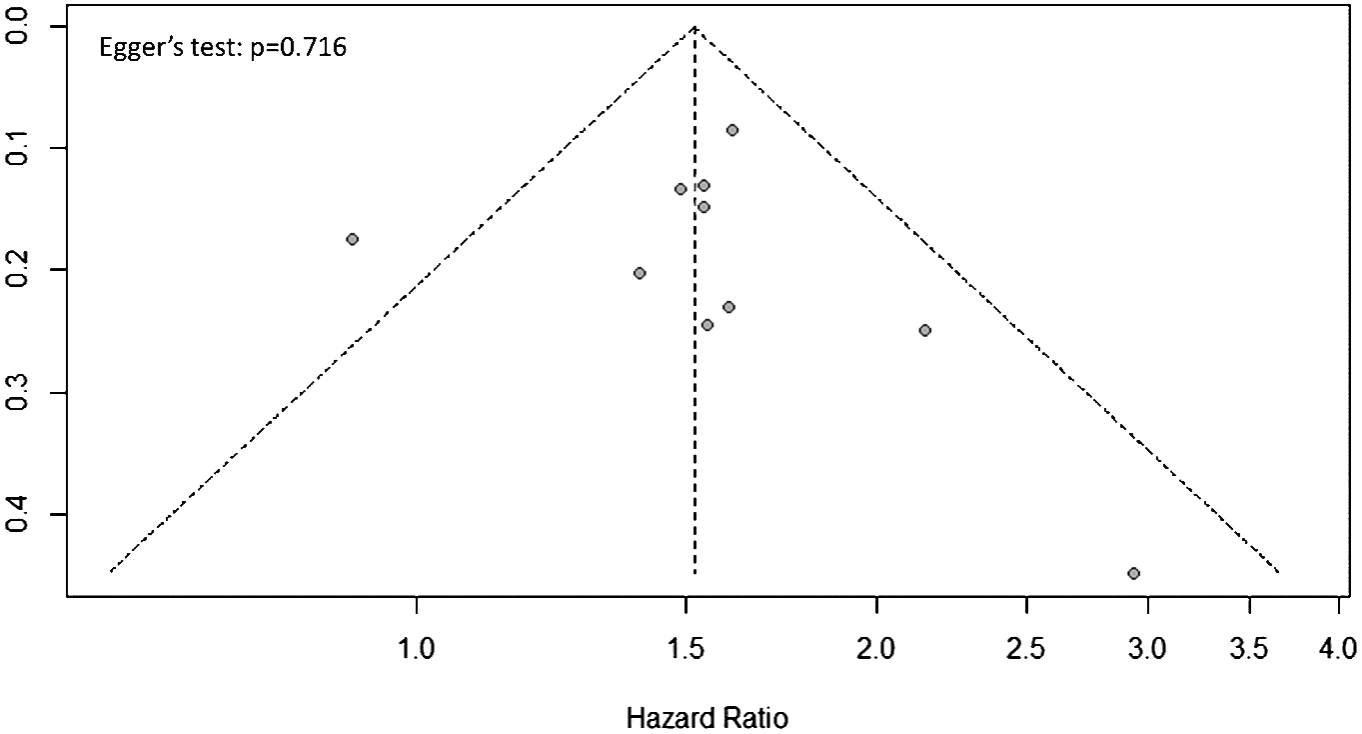
Funnel plots of publication bias for the studies assessing overall survival (OS).

Publication bias assessment should only be conducted for outcomes reported in 10 or more studies to assure the power of the test (26). In the present meta-analysis, the only outcome meeting this criterion is OS. The funnel plot for publication bias assessment of the studies reporting OS is shown in **Figure 4**. The data points in the funnel plot appears graphically symmetric, indicating there is no evidence of publication bias by Egger’s regression test (p = 0.716).

### Sensitivity analyses

The results of the sensitivity analysis using the leave-one-out approach are shown in **Table 2**. The direction and the magnitude of the effect size were not influenced by any single study. Nevertheless, when the study by van Vugt et al. (19) was excluded, the I^2^ value decreased to 0%, and the pooled HR for OS increased to 1.587 (95% CI, 1.435–1.755).

**Table 2:** Sensitivity analyses for OS (myosteatosis vs. no myosteatosis) through leave-one-out approach.

Study left out	HR (95% CI)	p-value	tau^2^	tau	I^2^
Chen et al. (24)	1.523 (1.373, 1.690)	< 0.001	0.006	0.079	40.20%
Koh et al. (25)	1.516 (1.368, 1.679)	< 0.001	0.005	0.07	40.30%
Kroenke et al. (16)	1.475 (1.311, 1.660)	< 0.001	0.007	0.084	37.00%
Park et al. (22)	1.526 (1.382, 1.686)	< 0.001	0.000	0.001	39.60%
Aro et al. (23)	1.517 (1.375, 1.674)	< 0.001	0.000	0.002	40.30%
Hopkins et al. (20)	1.515 (1.3652, 1.681)	< 0.001	0.007	0.082	40.30%
Aro et al. (21)	1.515 (1.372, 1.672)	< 0.001	0.000	0.019	40.10%
Sueda et al. (17)	1.507 (1.367, 1.660)	< 0.001	0.000	0.003	28.70%
van Baar et al. (18)	1.497 (1.357, 1.652)	< 0.001	0.000	0.001	29.70%
van Vugt et al. (19)	1.587 (1.435, 1.755)	< 0.001	0.000	0.001	0.00%
Pooled estimate	1.519 (1.379, 1.672)	< 0.001	0.000	0.001	32.90%

OS, overall survival; HR, hazard ratio; CI, confidence interval.

### Quality assessment

The quality ratings of each study using the NOS are shown in **Table 1**. The scores ranged from 6 to 8, indicating that the studies demonstrated a moderate to high level of quality.

## Discussion

This updated systematic review and meta-analysis, which included 9,203 patients with CRC in 10 studies undergoing curative surgery, was performed to synthesize the most current evidence regarding the impact of myosteatosis on the prognosis of CRC. The results revealed that myosteatosis was 1) associated with a 52% higher risk of poor OS, 2) a 67% increased likelihood of unfavorable CSS, and 3) an 89% greater risk of poor DFS. These findings indicate the importance of assessing myosteatosis in patients with CRC in order to evaluate risk and plan treatment and potential interventions to enhance patient outcomes and quality of life.

Myosteatosis, the infiltration of fat into muscle, has attracted increasing research over the past decade and has been associated with a number of diseases, including but not limited to the presence of dysglycemia, insulin resistance, and type 2 diabetes mellitus (27), worse outcomes in patients undergoing surgery for chronic pancreatitis (28), higher overall mortality in patients with cirrhosis (29), and higher complication rates in patients undergoing orthotopic liver transplantation (30). A recent, notably study, included routine abdominal computed tomography (CT) scans of approximately 9,000 outpatients (31). The CT scans were examined by an artificial-intelligence-based profiling of body composition. The results showed that in asymptomatic adults, myosteatosis was associated with a significantly increased risk of mortality (HR = 1.89; 95% CI, 1.52–2.35; p < 0.001).

A recent systematic review and meta-analysis examined the association between myosteatosis and the prognosis of various malignancies (4). The study included approximately 21,000 patients, and the results showed that myosteatosis was associated with significantly worse OS in patients with gynaecological, renal, pancreatic, hepatocellular, gastroesophageal, and colorectal cancers, and patients with lymphoma. While our study was focused on CRC, other studies have examined myosteatosis and the outcomes of gastric cancer and other digestive system malignancies (32–35). Fang et al. (34) performed a meta-analysis examining the impact of myosteatosis on the OS of patients with gastric cancers. The analysis included approximately 5,900 patients, and the results showed that myosteatosis was associated with a significantly increased mortality risk (HR = 1.46), and in the subgroup of patients undergoing surgery, it was associated with significantly shorter OS. Murnane et al. (32) studied 108 patients who received radical esophageal and gastric cancer surgery and found that myosteatosis was associated with a significantly increased risk of overall and severe complications, and reduced long-term survival.

Two recent meta-analyses have examined the association between myosteatosis and gastrointestinal malignancies. The study by MacCormick et al. (35) included approximately 14,500 patients with gastrointestinal malignancies who received surgery. Patients with myosteatosis had significantly poorer OS (HR = 1.66), CSS (HR = 1.73), and recurrence-free survival (HR = 1.38). Wang et al. (33) performed a meta-analysis to examine the association between myosteatosis and the OS of patients with digestive system malignancies. Digestive system malignancies included esophageal cancer, gastric cancer, CRC, hepatocellular cancer, pancreatic cancer, periampullary cancer, biliary tract cancer, cholangiocarcinoma, and mixed tumor type malignancies. Overall, patients with myosteatosis had a 44% increased mortality risk compared to patients without myosteatosis. However, subgroup analysis showed that the predictive value of myosteatosis for increased mortality risk was only significant for patients with esophageal cancer, cholangiocarcinoma/pancreatic cancer, and CRC.

The most relevant prior work in the literature is a systematic review and meta-analysis published in 2020, specifically examining the prognostic impact of myosteatosis in patients with CRC (13). That review included approximately 8,600 patients, and the results showed that patients with myosteatosis had a significantly increased overall mortality rate (HR = 1.55). Notably, as reported in that review, the negative impact of myosteatosis was independent of the coexistence of sarcopenia. Our current meta-analysis offers several advantages over the work of Lee and Kang. First, all the studies included in their analysis were published before 2020, while our analysis incorporates five studies published between 2020 and 2022. This inclusion provides a more contemporary reflection of the care scenario in recent years. Second, our findings indicate that myosteatosis is associated with worse DFS, contrasting with Lee and Kang’s report, which found no significant effect of myosteatosis on DFS. It is worth noting that their analysis on DFS seemed to mix univariate and multivariate results, potentially leading to their non-significant findings. In contrast, we only pooled studies that provided adjusted HRs, making our analysis more reliable than the prior meta-analysis.

Different from myosteatosis, sarcopenia is a widely recognized concept that encompasses the loss of muscle mass, quantity or quality, and low physical performance associated with aging. Sarcopenia has been linked with various malignancies, including digestive system cancers and is a predictor of poor long-term prognosis (36). Myosteatosis may be incorporated into the definition of sarcopenia, as it can reduce muscle function before there is a measurable loss of muscle mass (36). Studies have examined the effect of sarcopenia on the outcomes of various malignancies, including CRC. Trejo-Avila et al. (37) performed a systematic review and meta-analysis of the relation between sarcopenia and outcomes of patients with CRC. The analysis included about 19,000 patients, of which 37% had sarcopenia. Patients with sarcopenia had a significantly higher risk of total postoperative complications (OR = 1.84), severe complications (OR = 1.72), and postoperative mortality OR = 3.21), and higher risks of infections, cardiopulmonary complications, and prolonged LOS. Also notable, patients with sarcopenia had significantly worse OS, CSS, and DFS. Another recent systematic review and meta-analysis examined CT assessment of sarcopenia and OS in patients with CRC (38).

Taking together, the findings of the current systematic review and meta-analysis offer the latest insights derived from up-to-date literature, emphasizing the evidence surrounding myosteatosis in relation to CRC outcomes, extending beyond the scope of sarcopenia. Moreover, considering its notable clinical relevance in post-surgical prognosis and the overarching goal of improving patient care while optimizing long-term outcomes, the incorporation of myosteatosis assessments into the diagnostic protocol for risk stratification in CRC surgeries may be imperative.

### Strengths and limitations

The current meta-analysis has several notable strengths. First and foremost, it incorporated the most recent and comprehensive body of evidence concerning the prognostic significance of myosteatosis in patients undergoing surgery for CRC. Additionally, the collective sample size of the studies included was substantial, enhancing the statistical power of the analysis. It is important to note that we exclusively included studies that presented adjusted HRs for survival outcomes, thereby controlling for potential confounding variables. Remarkably, we observed minimal heterogeneity across the included studies, which suggests that our analytical results are reliable and robust. However, several limitations should be acknowledged. With the exception of one study, all others were retrospective in nature, potentially rendering them susceptible to selection bias. Additionally, owing to the limited availability of data, we were unable to perform a separate analysis stratified by tumor characteristics such as stage and primary site. This constraint could potentially introduce bias into the pooled findings, even though adjustments were made for these factors. In light of these limitations, and to further substantiate our conclusions, it is imperative that more prospective studies are conducted in the future.

## Conclusion

This updated systematic review and meta-analysis clearly demonstrates that myosteatosis is associated with significantly worse outcomes of patients undergoing surgery for CRC, including higher risks for poor OS, CSS, and DFS. These findings underscore the importance of assessing myosteatosis in patients with CRC to inform treatment decisions and improve overall outcomes.

## Data availability statement

The original contributions presented in the study are included in the article/supplementary material. Further inquiries can be directed to the corresponding author.

## Author contributions

Y-YC: Conceptualization, Data curation, Formal analysis, Investigation, Supervision, Writing – review & editing. BC: Data curation, Formal analysis, Investigation, Methodology, Project administration, Writing – original draft, Writing – review & editing.
